# Metabolites as Risk Factors for Diabetic Retinopathy in Patients With Type 2 Diabetes: A 12-Year Follow-up Study

**DOI:** 10.1210/clinem/dgad452

**Published:** 2023-08-10

**Authors:** Lilian Fernandes Silva, Jenna Hokkanen, Jagadish Vangipurapu, Anniina Oravilahti, Markku Laakso

**Affiliations:** Institute of Clinical Medicine, Internal Medicine, University of Eastern Finland, 70211 Kuopio, Finland; Institute of Clinical Medicine, Internal Medicine, University of Eastern Finland, 70211 Kuopio, Finland; Institute of Clinical Medicine, Internal Medicine, University of Eastern Finland, 70211 Kuopio, Finland; Institute of Clinical Medicine, Internal Medicine, University of Eastern Finland, 70211 Kuopio, Finland; Institute of Clinical Medicine, Internal Medicine, University of Eastern Finland, 70211 Kuopio, Finland; Department of Internal Medicine, Kuopio University Hospital, 70211 Kuopio, Finland

**Keywords:** diabetic retinopathy, type 2 diabetes, metabolomics, metabolite

## Abstract

**Context:**

Diabetic retinopathy (DR) is a specific microvascular complication in patients with diabetes and the leading cause of blindness. Recent advances in omics, especially metabolomics, offer the possibility identifying novel potential biomarkers for DR.

**Objective:**

The aim was to identify metabolites associated with DR.

**Methods:**

We performed a 12-year follow-up study including 1349 participants with type 2 diabetes (1021 without DR, 328 with DR) selected from the METSIM cohort. Individuals who had retinopathy before the baseline study were excluded (n = 63). The diagnosis of retinopathy was based on fundus photography examination. We performed nontargeted metabolomics profiling to identify metabolites.

**Results:**

We found 17 metabolites significantly associated with incident DR after adjustment for confounding factors. Among amino acids, N-lactoyl isoleucine, N-lactoyl valine, N-lactoyl tyrosine, N-lactoyl phenylalanine, N-(2-furoyl) glycine, and 5-hydroxylysine were associated with an increased risk of DR, and citrulline with a decreased risk of DR. Among the fatty acids N,N,N-trimethyl-5-aminovalerate was associated with an increased risk of DR, and myristoleate (14:1n5), palmitoleate (16:1n7), and 5-dodecenoate (12:1n7) with a decreased risk of DR. Sphingomyelin (d18:2/24:2), a sphingolipid, was significantly associated with a decreased risk of DR. Carboxylic acid maleate and organic compounds 3-hydroxypyridine sulfate, 4-vinylphenol sulfate, 4-ethylcatechol sulfate, and dimethyl sulfone were significantly associated with an increased risk of DR.

**Conclusion:**

Our study is the first large population-based longitudinal study to identify metabolites for DR. We found multiple metabolites associated with an increased and decreased risk for DR from several different metabolic pathways.

Diabetic retinopathy (DR) is a specific microvascular complication in patients with type 1 diabetes and type 2 diabetes (T2D), and the leading cause of blindness (1). A 10-year incidence of DR was 74% in the Wisconsin Epidemiologic Study of Diabetic Retinopathy, and about 20% of the patients with type 1 diabetes and 14% to 25% of patients with T2D developed macular edema (2). Risk factors for DR include hyperglycemia, hypertension, dyslipidemia, diabetes duration, and genetic factors (3, 4).

Chronic exposure to hyperglycemia and other causal risk factors initiates a cascade of biochemical and physiological changes that ultimately lead to microvascular damage and retinal dysfunction. The main findings in DR are hyperglycemia-induced pathological alterations, including oxidative stress, inflammation, angiogenesis, and accumulation of advance glycation end products (5-7), resulting in overactivation of protein kinase pathway, increased apoptosis of endothelial cells and neurons, and damage in the retinal blood capillaries (5, 8-9).

Recent advances in omics, especially metabolomics, offer the possibility to identify novel potential biomarkers for DR (10-16). Metabolomics can be performed on plasma, serum, vitreous humor, aqueous humor, retina, urine, and feces (17). The most important analytical technologies for metabolomics are mass spectrometry and nuclear magnetic resonance. Mass spectrometry is extensively applied in metabolomics studies combined with a chromatographic separation phase, such as liquid chromatography or gas chromatography-mass spectrometry (17).

Given the multiple sources of samples and different technologies applied in the measurement of metabolites makes it challenging to compare the results from different studies of DR given the fact that previous studies have included only a limited number of participants. In a systemic review of the studies on metabolomics in DR, Hou et al reported the results from 9 studies, having from 42 to 173 participants (18). Four of these studies reported increases in plasma levels of citrulline (19), L-glutamine (20), and acetic acid (21), and decreases in L-glutamic acid in patients with T2D with DR compared with patients with T2D without retinopathy. Multiple separate studies have been published, but the results published have not been replicated in other studies (19-23).

All previously published studies on metabolites associated with DR have been cross-sectional, which is the limitation of previous studies. We performed a 12-year follow-up study including 1349 T2D participants with DR (n = 328) and without DR (n = 1021) from the METSIM cohort, and identified several new metabolites associated with the risk of DR.

## Materials and Methods

### Design, Setting, and Study Population

The participants were selected from the METSIM study, comprising 10 197 Finnish men randomly selected from the population register of Kuopio, Eastern Finland, and aged from 45 to 73 years at baseline. We have previously described the design of this study (24). Our study included 1349 individuals with T2D. A total of 63 individuals had DR before the baseline study and they were excluded from all statistical analyses. The study was approved by the Ethics Committee of the Kuopio University Hospital (number: 174/2004; approval: 29 November 2004). All study participants gave written informed consent. We performed all laboratory methods, including metabolomics analysis, in accordance with the relevant guidelines and regulations.

### Clinical and Laboratory Measurements at Baseline

Height was measured without shoes to the nearest 0.5 cm. Weight was measured with a calibrated digital scale (Seca 877, Hamburg, Germany), and rounded up to the nearest 0.1 kg. Body mass index was calculated as weight (kg) divided by height (m) squared. The diagnosis of DR was based on fundus photography examination (dilated pupils, 1 field, 30 degrees). Ophthalmologists at the Kuopio University Hospital evaluated retinal microvascular findings, microaneurysms, macular edema, hemorrhages, soft and hard exudates, intraretinal microvascular abnormality, and laser treatment. They classified the findings as nonproliferative DR and proliferative DR needing laser treatment, depending on ophthalmologic changes and retinal neovascularization recorded in the medical records (25, 26).

Laboratory measurements after 12 hours of fasting have been previously described (27), and they included the measurements of glucose, hemoglobin A1c (HbA1c), total triglycerides, low-density lipoprotein (LDL) cholesterol, high-sensitivity C-reactive protein, and mass spectrometry metabolomics (Metabolon, Durham, NC, USA). An oral glucose tolerance test was performed (75 g of glucose) to evaluate glucose tolerance according to American Diabetes Association criteria (28). We measured glucose using an enzymatic hexokinase photometric assay (Konelab Systems Reagents, Thermo Fischer Scientific, Vantaa, Finland), and total triglycerides and LDL cholesterol using enzymatic colorimetric methods (Konelab Systems Reagents; Thermo Fisher Scientific, Vantaa, Finland).

### Metabolomics

Metabolomics analysis (Metabolon Inc., Durham, NC, USA) was used to perform nontargeted metabolomics profiling for the participants of the METSIM study at the baseline visit, as previously described in detail (29, 30). EDTA plasma samples were obtained after ≥10 hours of overnight fasting. After methanol extraction of biochemicals, nontargeted relative quantitative liquid chromatography-tandem mass spectrometry (Metabolon Discovery HD4 platform) was performed to identify named metabolites. A total of 1009 unique known metabolites were included in statistical analyses. The subclassification of the lipids was based on the Human Metabolome Database (http://www.hmdb.ca).

### Statistical Analysis

**Figure 1.**
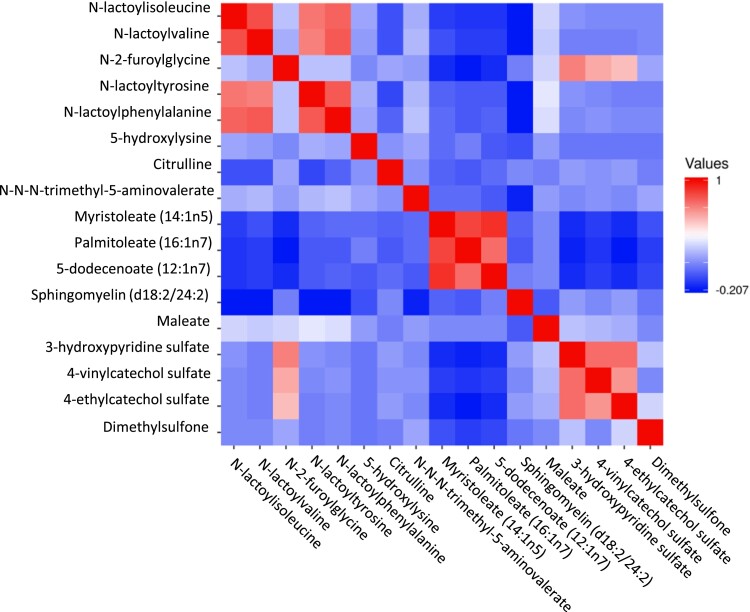
Heatmap showing the correlations between the metabolites associated with retinopathy.

Statistical analyses were performed using IBM SPSS Statistics 25. All variables were log-transformed to correct for their skewed distribution. In metabolite analyses, *P* < 5.0 × 10^−5^ was statistically significant given the 1009 metabolites measured. We applied analysis of variance for independent samples to compare the 2 groups (Table 1). Metabolites associated with DR were analyzed with a Cox regression model. The hazard ratio and 95% CI were calculated. The results are given unadjusted and adjusted for confounding factors (age, HbA1c, systolic blood pressure, and smoking) (Table 2). Correlations between the metabolites were calculated using Spearman correlations (Fig. 1).

**Table 1.** Comparison of the baseline characteristics of the participants having type 2 diabetes without retinopathy and with retinopathy

	No retinopathy			Retinopathy			*P*
	n	Mean	SE	n	Mean	SE	
Age, years	1021	60.3	0.21	328	60.8	0.36	.231
HbA1c, %	1021	6.34	0.03	328	7.06	0.07	**2.0 × 10^−28^**
Systolic blood pressure, mmHg	1020	144	0.56	328	148	1.02	.**003**
Current smoking, %	1021	1.13	0.03	328	1.25	0.05	.047*
Body mass index, kg/m^2^	1020	30.0	0.17	328	30.5	0.26	.185
Total triglycerides, mmol/L	1021	1.84	0.04	327	2.08	0.08	.**003**
Total cholesterol, mmol/L	1020	5.02	0.04	327	4.89	0.06	.061
LDL cholesterol, mmol/L	1021	3.02	0.03	327	2.90	0.05	.042
hs-CRP, mg/L	1021	3.45	0.21	328	2.60	0.24	.029
Estimated GFR (mL/min/1.73 m^2^)	1020	86.60	0.043	327	86.91	0.079	.729
Cardiovascular events, %	1020	16.6	—	327	17.5	—	.404*
Statin users, %	1020	45.8	—	327	52.9	—	.015*

Results obtained using analysis of variance.

*Chi-square test, statistically significant *P* value, *P* < .006 (bolded).

Abbreviations: GFR glomerular filtration rate; hs-CRP; high-sensitivity C-reactive protein; LDL, low-density lipoprotein.

**Table 2.** Metabolites associated with retinopathy during follow-up in participants with type 2 diabetes

Metabolite	Subclass	Cases	Controls	HR	Lower	Upper	*P*	P*	P**
**Amino acids**									
N-lactoyl isoleucine	N-lactosyl amino acids	319	985	1.35	1.24	1.46	**2.8 × 10^−13^**	5.6 × 10^−4^	.001
N-lactoyl valine	N-lactosyl amino acids	327	1018	1.36	1.25	1.48	**1.8 × 10^−12^**	2.6 × 10^−4^	2.3 × 10^−4^
N-lactoyl tyrosine	N-lactosyl amino acids	294	864	1.32	1.21	1.44	**2.5 × 10^−10^**	4.8 × 10^−4^	3.0 × 10^−4^
N-lactoyl phenylalanine	N-lactosyl amino acids	327	1019	1.28	1.19	1.39	**5.0 × 10^−10^**	.002	.002
N-(2-furoyl)glycine	N-acyl-alpha amino acids	304	917	1.31	1.22	1.42	**2.3 × 10^−12^**	2.1 × 10^−4^	3.6 × 10^−4^
5-Hydroxylysine	L-alpha-amino acids	326	1015	1.23	1.12	1.35	**1.0 × 10^−5^**	.002	.001
Citrulline	L-alpha-amino acids	327	1019	0.83	0.77	0.91	**1.8 × 10^−5^**	.017	.005
**Fatty acids**									
N,N,N-trimethyl-5-aminovalerate	Straight-chain fatty acids	327	1019	1.27	1.14	1.41	**7.5 × 10^−6^**	.004	.004
Myristoleate (14:1n5)	Long-chain MUFA	327	1019	0.79	0.72	0.88	**5.9 × 10^−6^**	4.2 × 10^−4^	**3.0 × 10^−5^**
Palmitoleate (16:1n7)	Long-chain MUFA	327	1019	0.81	0.73	0.89	**2.8 × 10^−5^**	2.4 × 10^−4^	**3.1 × 10^−5^**
5-dodecenoate (12:1n7)	Long-chain MUFA	327	1019	0.81	0.73	0.89	**2.6 × 10^−5^**	.001	1.4 × 10^−4^
**Sphingolipids**									
Sphingomyelin (d18:2/24:2)*	Sphingomyelin	327	1018	0.80	0.72	0.88	**1.1 × 10^−5^**	.015	.005
**Carboxylic acids**									
Maleate	Dicarboxylic acid	309	971	1.21	1.14	1.28	**6.4 × 10^−11^**	**4.0 × 10^−6^**	**5.6 × 10^−6^**
**Organic compounds**									
3-Hydroxypyridine sulfate	Arylsulfates	326	922	1.32	1.21	1.45	**4.3 × 10^−9^**	**1.6 × 10^−6^**	**2.3 × 10^−6^**
4-Vinylcatechol sulfate	Sulfated Catechol	294	922	1.31	1.19	1.44	**2.8 × 10^−8^**	**4.7 × 10^−7^**	**3.1 × 10^−7^**
4-Ethylcatechol sulfate	Sulfated catechols	322	1004	1.25	1.15	1,36	**3.9 × 10^−7^**	**4.5 × 10^−7^**	**2.9 × 10^−6^**
Dimethyl sulfone	Sulfone	323	991	1.25	1.15	1.36	**3.0 × 10^−7^**	**4.0 × 10^−8^**	**9.0 × 10^−7^**

Cases (in table) are participants who had retinopathy at follow-up alone. Results are based on unadjusted Cox regression analysis.

Abbreviation: MUFA, monounsaturated fatty acid.

*P* unadjusted, *P adjusted for HbA1c, **P adjusted for age, HbA1c, systolic blood pressure, and smoking at baseline. Mean follow-up was 11.7 years. Statistically significant *P* < .006 (bolded).

## Results

### Clinical and Laboratory Characteristics

Clinical and laboratory characteristics of the participants without DR (n = 1021) and the participants who developed retinopathy during the follow-up (DR, n = 328; 15 had proliferative DR and laser treatment, 313 had nonproliferative DR) having T2D are given in Table 1. These 2 groups did not differ significantly with respect to age, smoking, body mass index, total and LDL cholesterol, high-sensitivity C-reactive protein, estimated glomerular filtration rate, cardiovascular events, or statin use at the baseline study. Participants with DR had increased blood pressure, total triglycerides, and especially more elevated HbA1c compared with participants without DR. Additionally, participants with DR more often had insulin treatment (20.5%) than participants without DR (5.4%, *P* = 1.8 × 10^−14^).

### Metabolites Associated With DR

We found 17 metabolites associated with DR without adjustment for confounding factors; 7 of them were statistically significant (*P* < 5.0 × 10^−5^) and 10 nominally significant (*P* < .006) after the adjustment for confounding factors. Among N-acyl-alpha amino acids, N-lactoyl isoleucine, N-lactoyl valine, N-lactoyl tyrosine, and N-lactoyl phenylalanine were nominally associated with an increased risk of DR. An N-acyl-alpha-amino acid (N-(2-furoyl)glycine) and an L-alpha-amino acid (5-hydroxylysine) were nominally associated with an increased risk of DR, whereas L-alpha-amino acid citrulline was associated with a decreased risk of DR.

Long-chain monounsaturated fatty acids (MUFAs) myristoleate (14:1n5) and palmitoleate (16:1n7) were significantly associated and 5-dodecenoate (12:1n7) nominally associated with a decreased risk of DR. N,N,N-trimethyl-5-aminovalerate was nominally associated with an increased risk of DR. Sphingomyelin (d18:2/24:2), a sphingolipid, was significantly associated with a decreased risk of DR. Carboxylic acid maleate and organic compounds 3-hydroxypyridine sulfate, 4-vinylphenol sulfate, 4-ethylcatechol sulfate, and dimethyl sulfone were significantly associated with an increased risk of DR.

We found that when the adjustment was done only for HbA1c *P* values were very similar to those when the adjustment was done for all confounding factors, except for N-lactoyl isoleucine and 5-dodecenoate. This suggests that HbA1c has a major effect on metabolite concentrations.

Figure 1 shows Spearman correlations between 17 metabolites associated with DR. N-acyl-alpha amino acids (N-lactoyl isoleucine, N-lactoyl valine, N-lactoyl-tyrosine, and N-lactoyl phenylalanine) had high intercorrelation (from 0.70 to 0.85). N-(2-furoyl)glycine, 5-hydroxylysine, and citrulline did not correlate significantly with any of the metabolites. Long-chain MUFAs (myristoleate, palmitoleate, 5-dodecenoate) had high intercorrelations (>0.70). Similarly, organic compounds (3-hydroxypyridine sulfate, 4-vinylcatechol sulfate, 4-ethylcatechol sulfate, quinate) had high intercorrelations (>0.70). Among the metabolites associated significantly with DR, 5-hydroxylysine, and citrulline had low correlations (<0.20) with other metabolites.

## Discussion

DR is a frequent microvascular complication in patients with diabetes. The most important risk factor for DR is hyperglycemia, as shown in our study. HbA1c concentration was significantly higher and insulin treatment more frequent among the patients with DR than in the patients without DR.

Finding of biomarkers for DR is of great interest and importance. Previous cross-sectional studies have reported inconsistent results due to a small number of participants and metabolites measured, and different technologies applied to identify metabolites. Our 12-year follow-up study to identify metabolites associated with the risk of DR is the first large longitudinal study including 1349 participants with T2D. We found 13 metabolites significantly associated with an increased risk of DR, 4 N-lactoyl-amino acids, 1 N-acyl-alpha amino acid, 1 L-alpha-amino acid, 1 straight-chain fatty acid, 1 dicarboxylic acid, and 5 organic compounds.

**Figure 2.**
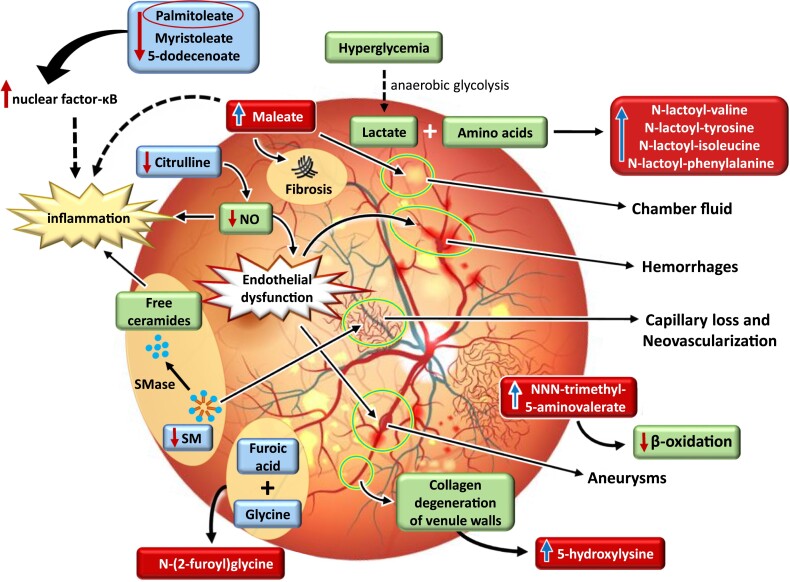
Metabolic changes in diabetic retinopathy that impair retina function. Abbreviations: NO, nitric oxide; SM, sphingomyelin; SMase, sphingomyelinase. In DR patients having hyperglycemia, glucose undergoes anaerobic glycolysis and generates lactate. To avoid deleterious effects of lactate on the retina, lactate is combined with amino acids to produce N-lactosyl amino acids. High levels of N, N, N-trimethyl-5-aminovalerate impair beta-oxidation in the retina. DR causes collagen degeneration of the venule walls by increasing 5-hydroxylysine levels. In the retina furoic acid, which is a toxic metabolite, is conjugated with glycine to generate a less toxic metabolite, N-(2-furoyl)-glycine. Sphingomyelin is a “reservoir” of ceramides, and its cleavage by sphingomyelinase generates free ceramides, which triggers inflammation. Low levels of sphingomyelin also contribute to capillary loss and neovascularization in DR. Low levels of citrulline in DR, lead to inflammation and to low levels of nitric oxide, causing endothelial dysfunction, which contributes to hemorrhages and aneurysms in the retina. Lower levels of MUFAs activate nuclear factor k-B, causing inflammation. Increased levels of maleate lead to inflammation, fibrosis, and chamber fluid vascular leakage in DR.

N-lactoyl-amino acids increased the risk of DR by 28% to 35%, which is a novel finding. N-lactoyl-amino acid generation in human plasma depends on lactate and amino acid concentrations (31). This interconversion happens quickly in living cells via the protease cytosolic nonspecific dipeptidase. Glucose is mostly metabolized into lactate by glycolysis rather than by oxidative phosphorylation in the retina (32). One possible mechanism could be that due to increased anaerobic glycolysis in participants with DR, lactate is incorporated into N-lactoyl-amino acids to prevent deleterious effects of lactate on glucose homeostasis (Fig. 2).

N-(2-furoyl) glycine, an N-acyl-alpha amino acid where glycine is conjugated with furoic acid, increased the risk of DR by 31%. This metabolite is a microbial metabolite and has not been previously associated with the risk of DR. It is involved in mitochondrial fatty acid beta-oxidation (33). Recent findings suggest that the glycine conjugation pathway is an essential detoxification pathway (34, 35). Glycine can be conjugated to various potentially toxic endogenous and xenobiotic metabolites. Conjugation forms of acylglycines are less toxic and excreted in urine (35).

5-Hydroxylysine, L-alpha-amino acid, increased the risk of DR by 23%, which is a novel finding. 5-Hydroxylysine is a marker of collagen degradation (33). A previous study investigated the changes in arteriole and venule in retina in advanced DR. When small retinal discs containing a precapillary arteriole and its corresponding postcapillary venule were examined by electron microscopy, the venule showed collagenous degeneration of the wall (36).

N,N,N-trimethyl-5-aminovalerate, a straight-chain fatty acid, increased the risk of DR by 27% in our study. This metabolite has not been previously associated with the risk of DR. N,N,N-trimethyl-5-aminovalerate, a degradation product of lysine or proline by gut microbiota, is a substrate for the cell membrane carnitine transporter and reduces cellular carnitine and β-oxidation of fatty acids (37-39) (Fig. 2). Lipid metabolism is important for retinal homeostasis, and the disruption of lipid entry into photoreceptors leads to extracellular lipid accumulation, suppressed glucose transporter expression, and a dual lipid/glucose fuel shortage (40). Of note, 5-aminovalerate, an upstream metabolite of N,N,N-trimethyl-5-aminovalerate, is absent in healthy controls but found in tear drops of DR patients (41).

Maleate, a dicarboxylic acid (33), increased the risk of DR by 21%. Several microbes convert maleic acid to D-malate by maleate hydratase enzyme (42). Intravitreal injection of malate into rabbits caused ocular irritation responses, including conjunctival redness, scleral swelling, chemosis, enlarged retinal blood vessels, and optic disc swelling, retinal folds, and retinal discoloration. Histopathologic evaluations revealed retinal degeneration, conjunctival inflammation, retinal pigment epithelial hypertrophy, optic nerve demyelination, anterior chamber fluid, and conjunctival fibrosis (43) (Fig. 2).

We found that participants with DR had significantly increased levels of 4 organic compounds. These organic compounds have not been previously associated with increased risk of DR. 3-Hydroxypyridine sulfate increased the risk of DR by 32%, 4-vinylcatechol sulfate by 31%, 4-ethylcatechol sulfate by 25%, and dimethyl sulfone by 25%. Adjustment for HbA1c did not change statistical significance, suggesting that an increased risk of DR attributable to organic compounds is largely independent of hyperglycemia, in contrast to all other metabolites that increased the risk of DR in our study. The mechanisms how organic compounds increase the risk of DR is not known.

We found that 5 metabolites were associated with a decreased risk of DR, amino acid citrulline (−17%), 3 MUFAs (myristoleate (14:1n5), −21%, palmitoleate (16:1n7) −19%, 5-dodecenoate (12:1n7) −19%), and sphingomyelin (d18:2/24:2), −30%). In agreement with our findings Alcubierre et al reported an inverse association of MUFAs with retinopathy (44). Palmitoleate controls adenosine monophosphate–activated protein kinase, resulting in a decrease in nuclear factor-κB and an increase in the expression of several anti-inflammatory factors (45). In contrast to our findings, 2 previous studies reported that citrulline increased the risk of DR (19, 22). Citrulline supplementation increases plasma arginine levels and leads to an increase in nitric oxide (NO) bioavailability (46-48) (Fig. 2). NO is a strong vasodilatory and anti-inflammatory signaling molecule that maintains vascular homeostasis and regulation of blood pressure. NO produced by endothelial cells is a critical regulator of this balance, such that endothelial dysfunction is defined as a reduced capacity for NO production and decreased NO sensitivity (49).

Sphingomyelins make up around 15% of the phospholipid content of human retina and protect the eye against oxidative damage (50). Acid sphingomyelinase, the enzyme that breaks down sphingomyelin into biological active ceramides, has been shown to be activated in the diabetic retina. Inhibition of this enzyme prevents inflammatory cytokine production, adhesion molecule expression, retinal capillary loss, and neovascularization in both vitro and in vivo models (51).

The strength of our study are that the METSIM study is a large randomly selected population-based cohort, and that we have detailed phenotype of 1349 participants including 1098 metabolites available. In addition, we used a very conservative threshold for statistical significance in our analyses of metabolites to increase credibility of our conclusions. The limitations of our study are that only middle-aged and elderly men were included, and that our study included only Finns. Therefore, our findings need to be replicated in women and other populations. Finally, our study is an association study that does not allow one to make causal conclusions.

In conclusion, our longitudinal study shows that the development of DR involves multiple changes in metabolism that ultimately impair retina function (Fig. 2). These changes in the retina, reflected by concentration changes in metabolites, include activation of inflammatory pathways, capillary loss, neovascularization, fibrosis, collagen degradation, endothelial dysfunction, hemorrhages, and aneurysms.

## Data Availability

Restrictions apply to the availability of data generated or analyzed during this study to preserve the confidentiality of the participants. The corresponding author will, on request, detail the restrictions and any conditions under which access to some data may be provided.

## Abbreviations

DR: diabetic retinopathy
HbA1c: hemoglobin A1c
LDL: low-density lipoprotein
MUFA: monounsaturated fatty acid
T2D: type 2 diabetes
